# Supplementary material for: A robust cusum control chart for median absolute deviation based on trimming and winsorization
Source: PLoS One. 2024 May 29;19(5):e0297544. doi: 10.1371/journal.pone.0297544 (PMC11135777; doi:10.1371/journal.pone.0297544)
Supplement: S1 Table — (DOCX) [file pone.0297544.s001.docx]

**Table1: R codes for standardized variation of robust estimators in different environments**

**Standard Deviation Code for Normal Distribution** $\mathbf{N(0,1)}$

x=c(); Mp=c(); Mn=c(); Q=c();Q1=c(); rl=c();UCL=c();

n=5;mu=0; sig=1; del=1;

k=1.08; h=1.531;

for(j in 1:1200)

{

for(i in 1:2000)

{

x[i]=sd(rnorm(n,0,del*sig));

#CUSUM Chart

if(i==1)

{Mp[i]=max(0,x[i]-k+0)}

else {Mp[i]=max(0,x[i]-k+Mp[i-1])}

if(Mp[i]>h)

{

rl[j]=Mp[i];

print(cbind(mean(rl),length(rl)))

break;}

else {rl[j]=0;}

}

}

res=cbind(del,k,h,median(rl),sd(rl)) # provide the results needed

res

sv <- n*var(rl)/(mean(rl))^2

sv
