# Supplementary material for: A robust cusum control chart for median absolute deviation based on trimming and winsorization
Source: PLoS One. 2024 May 29;19(5):e0297544. doi: 10.1371/journal.pone.0297544 (PMC11135777; doi:10.1371/journal.pone.0297544)
Supplement: S2 File — (DOCX) [file pone.0297544.s003.docx]

**R codes for ARLand SDRL**

**Table 5 & Table 6 : Standard Deviation Codes**

#----ARL & SDRL-----#

set.seed(1234)

sim0=20000

sy=Csump=Csumn=c(sim0)

nRL=12000

n=9

k=1.13

h=0.916

shft=3.00

nShft=length(shft); RL=c(nRL)

ARL=SDRL=MDRL=double(nShft)

mY0=Csump0=Csumn0=0; vY0=1; sY0=sqrt(vY0)

K=k*sY0; Hn=h*sY0

K=k*sY0; Hp=h*sY0

# =============================

# RL Calculations for all Shift

# =============================

mY1=mY0; sY1=shft*sY0

for(I in 1:nRL) #-------------- Start of RL loop --------------

{

for(i in 1:sim0) #----- start of sim loop -----

{y=rnorm(n, mY0, sY1)

sy[i]=sd(y)

if(i==1)

{Csump[i]=max(0, (sy[i]-K+Csump0))

Csumn[i]=min(0, (sy[i]+K+Csumn0))}

else

{Csump[i]=max(0, (sy[i]-K+Csump[i-1]))

Csumn[i]=min(0, (sy[i]+K+Csumn[i-1]))}

if(Csumn[i] < -Hn | Csump[i] > Hp)

{RL[I]=i;break}

} #----- end of sim loop -----

} # -------------- End of RL loop --------------

ARL =mean(RL)

SDRL=sd(RL)

MDRL=median(RL)

ARL

SDRL

MDRL
