# Supplementary material for: A robust cusum control chart for median absolute deviation based on trimming and winsorization
Source: PLoS One. 2024 May 29;19(5):e0297544. doi: 10.1371/journal.pone.0297544 (PMC11135777; doi:10.1371/journal.pone.0297544)
Supplement: Attachment — Submitted filename: PONE-D-23-01321_Reviewers comments.pdf [file pone.0297544.s004.pdf]

We have completed the review of your manuscript. When revising your manuscript, please consider all issues mentioned in the reviewers' comments, outline every change made in response to their comments and provide suitable rebuttals for any comments not addressed.

Comments
Reviewer 1:

This paper propose a robust CUSUM chart to evaluate the performance of dispersion parameters. The design structure features of various control charts, based on currently defined estimators and some new robust scale estimators by using trimming and winsorization in different scenarios, are examined. The Median Absolute Deviation based on trimming and winsorization is introduced. The performance of the new proposed chart are evaluated in terms of ARL and SDRL.

After addressing the points below, I would recommend the paper for publication.

1. Page 7, in the Introduction, paragraph 1, the abbreviation of " EWMA" should not be used when it is used for the first time. It should be " Exponentially Weighted Moving Average (EWMA)".
2. Page 8, Line 8: Change " WANG et al. [17]" to " Wang et al. [17]". Please check the format of the references throughout the paper. There are many mistakes.
3. Page 9, in the last paragraph: Change " Tables 1 and Tables 2" to " Table 1 and Table 2".
4. Page 11, in the first paragraph: Change " distribution-free" to " Distribution-free" and change " Amitava Mukherjee & Marco Marozzi [35]" to " Amitava and Marco [35]".
5. Page 11, in the title of Table 5, please change " Charts" to " charts". The same problem can be found in Tables 6-14.
6. Page 12, in the first paragraph, change " Table 6 show that" to "Table 6 shows that".
7. Page 14, in Section 6, please change " evaluated;" to "evaluated.".
8. In many Tables, when $\delta = 1$ and $\alpha = 0$, that is to say, when the process is in control and there is no contaminated data, why the in control ARL, i.e., $ARL0$ is not equal to 500?
9. Although I am not a native English speaker, but I found a number of ambiguous sentences typos, and spelling mistakes. The presentation and writing of the paper should be improved.
10. The references in page 14-17 are so badly presented in the paper. The format of the reference is inconsistent. I never seen such careless authors before. There are many such kind of mistakes in the paper.

Reviewer 2:
Comments:

The authors of this paper considers several robust estimators for the CUSUM chart. The performance measures of ARL and SDRL are used to evaluate the performance of the proposed estimators. The paper contains some merit, however, this paper is not well written and cannot be accepted for publication in its present form. This paper needs a major revision and its English must be significantly improved especially for Sections 3, 4 and 5. The following are the issues that the authors must address:

Major comments:

1. Section 1, paragraphs 2, 3, 4 and 5: Rewrite the literature review by adding more relevant information and discuss the results/ drawback from the existing methods. Writing a literature review is not just summarizing the existing works but you have to organize it in a way that we can see the overview of the works that have been proposed long time ago and have been improved over time until today. In addition, a thorough review of claiming the simplicity and efficiency of the CUSUM chart and the benefits for using the non-parametric charts are missing from the current paper. The novelty of the paper is not highlighted.
2. Section 4: (i) Define ARL_0 . In addition, the term out-of-control ARL (ARL_1) and SDRL ($SDRL_1$) should be used when the shift size, δ > 0. Avoid using the general term ARL (SDRL) as it comprises of ARL_0 ($SDRL_0$) when the process is in-control, and $ARL1$ ($SDRL_1$) when the process is out-of-control. (ii) Add in a section to explain the steps in constructing the proposed chart by using Monte Carlo simulation, so that the computation of out-of-control ARL (ARL_1) will be clearer. (iii) Give numerical examples while explaining the obtainment of ARL_0 value in Tables 3 and 4. As in current write-up, it is not clear whether $H_{\widehat{\theta}}$ or $K_{\widehat{\theta}}$ has to be searched until the desired ARL_0 is obtained or both $H_{\widehat{\theta}}$ and $K_{\widehat{\theta}}$ have to be searched until the desired ARL_0 is obtained. It seems like ARL_0 is affected by $K_{\widehat{\theta}}$ with unknown $H_{\widehat{\theta}}$ (Table 3) or affected by $H_{\widehat{\theta}}$ with unknown $K_{\widehat{\theta}}$ (Table 4) value.
3. Sections 5.1.1, 5.1.2, 5.1.3 and 5.1.4: Rewrite the analysis as there are (i) no explanation on why the robust estimator is claimed to be best performer, the ARL value has to be large or small etc? Give numerical example to depict the trend as well. (ii) no explanation for the SDRL values across different estimators. What does it mean to have large value in ARL/SDRL in Tables 5-14, perhaps this can be explained further.
4. Show the derivations of Equations (2), (5) and (8).

Minor Comments

1. Check the font's colour, theme fonts and indent of paragraph used throughout the paper. A number of inconsistencies were detected in the paper.
2. Abstract, line 2 up:….in observing small magnitude changes to both normal and contaminated data.
3. Abstract and Section 5: Add in the discussion to highlight whether the proposed estimators outperform existing estimators in literature review.
4. Section 1, last paragraph: "The purpose of this paper is to alter the use of dispersion parameters in CUSUM control…..for higher efficiency." should be removed to previous paragraph.
5. Could you add in the explanation on why the upper sided CUSUM chart is selected? The lower sided CUSUM will result in the same observations as well?
6. Section 2, line 8: Revise "The following estimators ……., which is described:" to "Two stimators are used in this paper. The first estimator of ϑ is the ….defined as (add in citation) :
7. Section 2, last paragraph: It should be sample Inter Quartile Range or sample interquartile range? Please standardize it.

8. Section 3, first paragraph, last line: "The trimmed mean ($\bar{Y}_T$) is defined as:". Do it similarly for Equations (4-8) by introducing the abbreviation before apply it.
9. The abbreviation MADM used in Tables and text is not defined.
10. Section 3: (i) It would be helpful to cite references that provide the Equations 1 – 8. (ii) Discuss Table 2 for $RE_{\hat{\theta}}$ (not $SV_{\hat{\theta}}$) results and relate it with Table 1. Thus, line 6 below Equation (10): It should be "Table 1 shows that the S estimator has the largest $SV_{\hat{\theta}}$ (i.e., 0.3690) under uncontaminated normal scenario, but it has……when sample size n = 9.
11. Section 3, Equation (9), line 2 up: …by Abbasi and Miller [29] are calculated.
12. Section 3: line 1 below Equation (10): "..$SV_{\hat{\theta}}$ and $RE_{\hat{\theta}}$ of robust estimators are examined and compared with several new robust estimators….." This sentence is confusing. I think you are referring to "the $SV_{\hat{\theta}}$ and $RE_{\hat{\theta}}$ values for all robust estimators are computed and compared."
13. Table 1, first row: Drop "δ " and replace it with "estimators"
14. The margin for Equation (6) is not consistent with others.
15. Section 4, Table 3, line 3 up: Table 3 and Table 4…. (i.e., normal and non-normal). distribution-free CUSUM. ..Incomplete sentences, these two sentences are confusing.
16. Section 5, paragraph 1, line 1: It is incorrect to mention ARL is used as an optimization method since you are using simulation method, not optimization formulae.
17. Check the margin for the numbering of the references.
