# Supplementary material for: A robust cusum control chart for median absolute deviation based on trimming and winsorization
Source: PLoS One. 2024 May 29;19(5):e0297544. doi: 10.1371/journal.pone.0297544 (PMC11135777; doi:10.1371/journal.pone.0297544)
Supplement: Attachment — Submitted filename: Referee Report_R1.pdf [file pone.0297544.s006.pdf]

Referee's Report

Journal : PLOS ONE
Title : A Robust CUSUM Control Chart for Median Absolute Deviation based on Trimming and Winsorization
Manuscript ID : PONE-D-23-01321R1
Date : June 9, 2023

Summary

The authors of this paper have addressed most of the comments raised in my previous report. However, there are still issues that must be addressed before the manuscript can be accepted for publication. The following are the issues that the authors must address from the manuscript (without track):

1. The English in this paper is not sufficiently improved. There are still grammatical mistakes spotted throughout the paper. For examples,

Section 1, paragraph 7, line 2: Wang et al. [22] introduced
Section 1, paragraph 8, line 6: Rahman et al [26] investigated
Abstract, line 2 up:….in observing small changes in magnitude for both normal and contaminated data.
Section 5.1.3, line 3: Table 9 and Table 10 show the results of ARL and SDRL respectively, for..
.
.
.
and others.

2. Section 1, paragraph 2: Remove "." in paragraph 2 and indent the first line.

3. Section 1, paragraph 7: Section 1, paragraph 7: Paragraph 7 should be indented.

4. Section 1: The paragraph's indentation and the spacing between paragraphs are not consistent. Please check.

5. Section 1, paragraph 7, line 3: …An improvement in process variation may lead to a rise in the number of defective products while a reduction in the process variability may add more things back to the goal…*The meaning of this phrase is not clear. Rephrase it. An improvement in process variation could indicate lower variability, thus it should reduce the number of defective products. In addition, what do you meant by add more things back? Avoid using the word "things", be clear and precise.*

6. Section 1, last second paragraph, line 5: The abbreviation SDRL is not defined in the text.

7. Section 1, last second paragraph, line 5: The purpose of this paper is to modify the use of dispersion parameters in the CUSUM control chart…

8. Add in citation for Equation 3 to justify why 1.4826 is selected.

9. Section 3, first paragraph, last line: Introduce the abbreviations before apply it. For examples,

"The trimmed mean ($\bar{Y}_T$) is defined as:",
"Mean deviation from trimmed mean (MDTM) is defined as:",
"Median of the absolute deviations from the trimmed mean (MADTM), which is defined as:"
Do it similarly for Equations (7-9).

10. Section 3, line below Equation (11): ..computed and compared.. Duplicated full stop.

11. Table 1: First row, the word "estimators" should be used instead of "δ " ?.

12. Section 5: Please change ARL to ARL_1 and SDRL to $SDRL_1$ in the discussion if $\delta > 1$ is highlighted. For example,

Section 5.1, line 4: ARL_1 increases when the process shift decreases.

13. Sections 5.1.1, 5.1.2, 5.1.3 and 5.1.4: Lack of explanation on why the robust estimator is claimed to be the best/ efficient performer. The analysis parts can be enhanced by giving some numerical values (obtained from the Table) to support the statement, so that the explanation will be clearer.

14. I think the authors didn't do the formatting for the references properly. A number of inconsistencies were detected in the paper. For examples,

a) "and" should be used or shouldn't be used when more than two authors,
b) full stop/ Comma placed wrongly,
c) the initial of given name(s) should come first or surname?
d) some with space between given name(s), some don't have space

15. The reference # [4] should be "S.W Roberts" not "G.C Roberts".
