# Supplementary material for: A robust cusum control chart for median absolute deviation based on trimming and winsorization
Source: PLoS One. 2024 May 29;19(5):e0297544. doi: 10.1371/journal.pone.0297544 (PMC11135777; doi:10.1371/journal.pone.0297544)
Supplement: Attachment — Submitted filename: Rebuttal letter plos one.docx [file pone.0297544.s005.docx]

**Rebuttal letter for revision of paper to PLOS ONE journal**

Dr. Umair Khalil
Academic Editor PLOS ONE & Associate Professor
Department of Statistics,

Abdul Wali Khan University Mardan, PAkistan

March 06, 2023

Dear Dr. Kok Haur Ng
Academic Editor
PLOS ONE

We are thankful to the reviewers for their valuable comments and suggestions on our manuscript title “A Robust Cusum Control Chart for Median Absolute Deviation based on Trimming and Winsorization”. We have revised the manuscript to address their comments.

The point to point response to the comments of reviewer 1 is as under:

Comments of Reviewer 1

Reviewer #1:

1. Page 7, in the Introduction, paragraph 1, the abbreviation of “ EWMA” should not be used when it is used for the first time. It should be “ Exponentially Weighted Moving Average (EWMA)”.

2. Page 8, Line 8: Change “ WANG et al. [17]” to “ Wang et al. [17]”. Please check the format of the references throughout the paper. There are many mistakes.

3. Page 9, in the last paragraph: Change “ Tables 1 and Tables 2” to “ Table 1 and Table 2”.

4. Page 11, in the first paragraph: Change “ distribution-free” to “ Distribution-free” and change “ Amitava Mukherjee & Marco Marozzi [35]” to “ Amitava and Marco [35]”.

5. Page 11, in the title of Table 5, please change “ Charts” to “ charts”. The same problem can be found in Tables 6-14.

6. Page 12, in the first paragraph, change “ Table 6 show that” to “Table 6 shows that”.

7. Page 14, in Section 6, please change “ evaluated;” to “evaluated.”.

8. In many Tables, when δ = 1 and α = 0, that is to say, when the process is in control and there is no contaminated data, why the in control ARL, i.e., ARL0 is not equal to 500?

9. Although I am not a native English speaker, but I found a number of ambiguous sentences typos, and spelling mistakes. The presentation and writing of the paper should be improved.

10.The references in page 14-17 are so badly presented in the paper. The format of the reference is inconsistent. I never seen such careless authors before. There are many such kind of mistakes in the paper

Response to reviewer 1

1. Page 7, in the Introduction, paragraph 1, the abbreviation of “ EWMA” “ Exponentially Weighted Moving Average (EWMA)” . It has been corrected.

2. Page 8, Line 8: Change “ WANG et al. [17]” to “ Wang et al. [17]” . It has been corrected.
3. Page 9, in the last paragraph: Change “ Tables 1 and Tables 2” to “ Table 1 and Table 2”. (corrected)
4. Page 11, in the first paragraph: Change “ distribution-free” to “ Distribution-free” and change “ Amitava Mukherjee & Marco Marozzi [35]” to “ Amitava and Marco [35]”. (corrected)
5. Page 11, in the title of Table 5, please change “ Charts” to “ charts”. The same problem can be found in Tables 6-14. (Corrected)
6. Page 12, in the first paragraph, change “ Table 6 show that” to “Table 6 shows that”.(Corrected)

7. Page 14, in Section 6, please change “ evaluated;” to “evaluated.”. (Corrected)
8. In many Tables, when δ = 1 and α = 0, that is to say, when the process is in control and there is no contaminated data, why the in control ARL, i.e., ARL0 is not equal to 500? (500 is targeted value not exact value)
9. The presentation and writing of the paper have been improved.
10. The references in page 14-17 have been corrected.

**The point to point response to the major comments of reviewer 2 is as under:**

Comments of Major Reviewer 2

Reviewer # 2:

1. Section 1, paragraphs 2, 3, 4 and 5: Rewrite the literature review by adding more relevant information and discuss the results/ drawback from the existing methods. Writing a literature review is not just summarizing the existing works but you have to organize it in a way that we can see the overview of the works that have been proposed long time ago and have been improved over time until today. In addition, a thorough review of claiming the simplicity and efficiency of the CUSUM chart and the benefits for using the non-parametric charts are missing from the current paper. The novelty of the paper is not highlighted.
2. Section 4: (i) Define 𝐴𝑅𝐿0 . In addition, the term out-of-control ARL (𝐴𝑅𝐿1) and SDRL (𝑆𝐷𝑅𝐿1) should be used when the shift size, 𝛿 > 0. Avoid using the general term ARL (SDRL) as it comprises of 𝐴𝑅𝐿0 (𝑆𝐷𝑅𝐿0) when the process is in-control, and 𝐴𝑅𝐿1 (𝑆𝐷𝑅𝐿1) when the process is out-of-control. (ii) Add in a section to explain the steps in constructing the proposed chart by using Monte Carlo simulation, so that the computation of out-of-control ARL (𝐴𝑅𝐿1) will be clearer. (iii) Give numerical examples while explaining the obtainment of 𝐴𝑅𝐿0 value in Tables 3 and 4. As in current write-up, it is not clear whether 𝐻𝜃̂ or 𝐾𝜃̂ has to be searched until the desired 𝐴𝑅𝐿0 is obtained or both 𝐻𝜃̂ and 𝐾𝜃̂ have to be searched until the desired 𝐴𝑅𝐿0 is obtained. It seems like 𝐴𝑅𝐿0 is affected by 𝐾𝜃̂ with unknown 𝐻𝜃̂ (Table 3) or affected by 𝐻𝜃̂ with unknown 𝐾𝜃̂ (Table 4) value.
3. Sections 5.1.1, 5.1.2, 5.1.3 and 5.1.4: Rewrite the analysis as there are (i) no explanation on why the robust estimator is claimed to be best performer, the ARL value has to be large or small etc? Give numerical example to depict the trend as well. (ii) no explanation for the SDRL values across different estimators. What does it mean to have large value in ARL/SDRL in Tables 5-14, perhaps this can be explained further.
4. Show the derivations of Equations (2), (5) and (8).

Response to Major Reviewer 2

1. Section 1, paragraphs 2, 3, 4, and 5: In paragraph 2,3, and 4 more information related to CUSUM charts have been added, new references has been added in this section in references 8, 9, 10, 11. The nonparametric chart is discussed in paragraph 5.
2. ARL_O_ has been defined in section 4. Flowchart has been constructed for the proposed method CUSUM charts in section 5, which explains the stepwise procedure for the process.
3. ARL and SDRL explanations are given in sections 5.1.1,5.1.2,5.1.3 and 5.1.4 in terms of shift 𝛿 operators from where it has been explained how the robust estimator performs well.
4. $MADTM =1.4826 Median \left| Y_{i}- \bar{Y}_{T} \right|.$ and $MADWM =1.4826 Median \left| Y_{i}- \bar{Y}_{w} \right|.$

In section 2: equation 2 is old estimator, in equation 3 $(MADM = Median \left| Y_{i}- \tilde{Y} \right|)$we replace sample median by trimmed mean and winsorizied mean to modify the new method for robust results as a proposed estimator.

The point to point response to the minor comments of reviewer 2 is as under:

Comments of Minor Reviewer 2

Reviewer # 2:

1. Se Check the font’s colour, theme fonts and indent of paragraph used throughout the paper. A number of inconsistencies were detected in the paper.
2. Abstract, line 2 up:….in observing small magnitude changes to both normal and contaminated

data.

3. Abstract and Section 5: Add in the discussion to highlight whether the proposed estimators

outperform existing estimators in literature review.

4. Section 1, last paragraph: “The purpose of this paper is to alter the use of dispersion

parameters in CUSUM control…..for higher efficiency.” should be removed to previous

paragraph.

5. Could you add in an explanation on why the upper sided CUSUM chart is selected? The lower

sided CUSUM will result in the same observations as well?

6. Section 2, line 8: Revise “The following estimators ……., which is described:” to “Two estimators are used in this paper. The first estimator of 𝜗 is the ….defined as (add in citation) :
7. Section 2, last paragraph: It should be sample Inter Quartile Range or sample interquartile range? Please standardize it.
8. Section 3, first paragraph, last line: “The trimmed mean (𝑌̅ 𝑇) is defined as:”. Do it similarly for

Equations (4-8) by introducing the abbreviation before apply it.

9. The abbreviation MADM used in Tables and text is not defined.
10. Section 3: (i) It would be helpful to cite references that provide the Equations 1 – 8. (ii) Discuss

Table 2 for 𝑅𝐸𝜃̂ (not 𝑆𝑉𝜃̂ ) results and relate it with Table 1. Thus, line 6 below Equation (10):

It should be “Table 1 shows that the S estimator has the largest 𝑆𝑉𝜃̂ (i.e., 0.3690) under

uncontaminated normal scenario, but it has……when sample size n = 9.

11. Section 3, Equation (9), line 2 up: …by Abbasi and Miller [29] are calculated.
12. Section 3: line 1 below Equation (10): “..𝑆𝑉𝜃̂ and 𝑅𝐸𝜃̂ of robust estimators are examined and

compared with several new robust estimators…..” This sentence is confusing. I think you are

referring to “the 𝑆𝑉𝜃̂ and 𝑅𝐸𝜃̂ values for all robust estimators are computed and compared.”

13. Table 1, first row: Drop “𝛿 ” and replace it with “estimators”
14. The margin for Equation (6) is not consistent with others.
15. Section 4, Table 3, line 3 up: Table 3 and Table 4…. (i.e., normal and non-normal). Distribution free CUSUM. ..Incomplete sentences, these two sentences are confusing.
16. Section 5, paragraph 1, line 1: It is incorrect to mention ARL is used as an optimization method

since you are using simulation method, not optimization formulae.

17. Check the margin for the numbering of the references

Response to Minor Reviewer 2

1. The inconsistencies has been removed
2. Abstract has been corrected.
3. Abstract and Section 5: It has been corrected and explanation has been added why it is better.
4. Section 1, last paragraph: It has been corrected.
5. In paragraph 8 explanation is given
6. Section 2, line 8: Revised
7. Section 2, last paragraph: “sample interquartile”. It has been corrected.
8. Section 3, equations (4-8).(Corrected)
9. The MADM is defined on page 3. (Corrected)
10. Section 3: It has been corrected.
11. Section 3, It has been corrected.
12. Section 3: line 1 below Equation (10): It has been corrected
13. Table 1, first row: Drop “𝛿 ” and replace it with “estimators”
14. The margin for Equation (6) has been corrected.
15. Section 4, Table 3, line 3 up: It has been corrected.
16. Section 5, paragraph 1, line 1: It has been corrected.
17. The margin for the numbering of the reference section 1, paragraphs 2, 3, 4 and 5 have been corrected.

Thank you for your consideration of this manuscript.

Sincerely,

Dr. Umair Khalil
