# Supplementary material for: A robust cusum control chart for median absolute deviation based on trimming and winsorization
Source: PLoS One. 2024 May 29;19(5):e0297544. doi: 10.1371/journal.pone.0297544 (PMC11135777; doi:10.1371/journal.pone.0297544)
Supplement: Attachment — Submitted filename: Second Rebuttal letter.docx [file pone.0297544.s007.docx]

**Second Rebuttal letter for Second revision of paper to PLOS ONE journal**

Dr. Umair Khalil
Academic Editor PLOS ONE & Associate Professor
Department of Statistics,

Abdul Wali Khan University Mardan, Pakistan

June 27, 2023

Dear Dr. Kok Haur Ng
Academic Editor
PLOS ONE

We are thankful to the reviewers for their valuable comments and suggestions on our manuscript title “A Robust Cusum Control Chart for Median Absolute Deviation based on Trimming and Winsorization”. We have revised the manuscript to address their comments.

The point to point response to the comments of reviewer 2 is as under:

| S.No | Comments of the reviewer | Response |
| --- | --- | --- |
| 01 | 1. The English in this paper is not sufficiently improved. There are still grammatical mistakes spotted throughout the paper. For examples,   Section 1, paragraph 7, line 2: Wang et al. [22] introduced  Section 1, paragraph 8, line 6: Rahman et al [26] investigated  Abstract, line 2 up:….in observing small changes in magnitude for both normal and contaminated data.  Section 5.1.3, line 3: Table 9 and Table 10 show the results of ARL and SDRL respectively, for.. | Section 1, paragraph 7 and 8 corrected  Abstract line 2 up.. has been corrected as suggested  Section 5.1.3, line 3 has been corrected |
| 02 | 1. Section 1, paragraph 2: Remove “.” in paragraph 2 and indent the first line. | It has been Indented |
| 03 | 1. Section 1, paragraph 7: Section 1, paragraph 7: Paragraph 7 should be indented. | It has been indented |
| 04 | Section 1: The paragraph’s indentation and the spacing between paragraphs are not consistent. Please check | it has been corrected |
| 05 | 1. Section 1, paragraph 7, line 3: …An improvement in process variation may lead to a rise in the number of defective products while a reduction in the process variability may add more things back to the goal…*The meaning of this phrase is not clear. Rephrase it. An improvement in process variation could indicate lower variability, thus it should reduce the number of defective products. In addition, what do you meant by add more things back? Avoid using the word “things”, be clear and precise.* | The suggested sentence has been rephrased as suggested |
| 06 | Section 1, last second paragraph, line 5: The abbreviation SDRL is not defined in the text | SDRL has been defined |
| 07 | Section 1, last second paragraph, line 5: The purpose of this paper is to modify the use of dispersion parameters in the CUSUM control chart | Section 1, last second paragraph has been Corrected as suggested |
| 08 | 1. Add in citation for Equation 3 to justify why 1.4826 is selected. | Explanation has been added for the value 1.4826 |
| 09 | Section 3, first paragraph, last line: Introduce the abbreviations before apply it. For examples,   1. “The trimmed mean (𝑌̅𝑇) is defined as:”,   “Mean deviation from trimmed mean (MDTM) is defined as:”,  “Median of the absolute deviations from the trimmed mean (MADTM), which is defined as:” Do it similarly for Equations (7-9). | All abbreviations has been introduced |
| 10 | Section 3, line below Equation (11): ..computed and compared.. Duplicated full stop. | Duplicate full stop has been removed |
| 11 | 1. Table 1: First row, the word “estimators” should be used instead of “𝛿 ” ?. | Table 1 corrected as suggested |
| 12 | 1. Section 5: Please change ARL to 𝐴𝑅𝐿_1_ and SDRL to 𝑆𝐷𝑅𝐿_1_ in the discussion if 𝛿 > 1 is highlighted. For example,   Section 5.1, line 4: 𝐴𝑅𝐿_1_ increases when the process shift decreases | Section 5 has been corrected as suggested |
| 13 | 1. Sections 5.1.1, 5.1.2, 5.1.3 and 5.1.4: Lack of explanation on why the robust estimator is claimed to be the best/ efficient performer. The analysis parts can be enhanced by giving some numerical values (obtained from the Table) to support the statement, so that the explanation will be clearer. | The larger the value of ARL for an in-control process is high, it has been highlighted in all the tables. Statement has been added in the suggested sections |
| 14 | 1. I think the authors didn’t do the formatting for the references properly. A number of inconsistencies were detected in the paper. For examples,    a) “and” should be used or shouldn’t be used when more than two authors,    b) full stop/ Comma placed wrongly,    c) the initial of given name(s) should come first or surname?    d) some with space between given name(s), some don’t have space | All the suggested corrections has been incorporated |
| 15 | 1. The reference # [4] should be “S.W Roberts” not “G.C Roberts”. | The reference [4] has been corrected |
