# Supplementary material for: A robust cusum control chart for median absolute deviation based on trimming and winsorization
Source: PLoS One. 2024 May 29;19(5):e0297544. doi: 10.1371/journal.pone.0297544 (PMC11135777; doi:10.1371/journal.pone.0297544)
Supplement: Attachment — Submitted filename: third Rebuttal letter.docx [file pone.0297544.s008.docx]

**Third Rebuttal letter for the Third revision of paper to PLOS ONE Journal**

Dr. Umair Khalil
Academic Editor PLOS ONE & Associate Professor
Department of Statistics,

Abdul Wali Khan University Mardan, Pakistan

August 21, 2023

Dear Dr. Kok Haur Ng
Academic Editor
PLOS ONE

We are thankful to the reviewers for their valuable comments and suggestions on our manuscript title “A Robust Cusum Control Chart for Median Absolute Deviation based on Trimming and Winsorization”. We have revised the manuscript to address their comments.

The point to point response to the comments of reviewer 2 is as under:

| S.No | Comments of the reviewer | Response |
| --- | --- | --- |
| 01 | Section 1: Instead of discussing the reviews one by one in different paragraphs, the authors should combine those reviews into the same paragraph. | Section 1, all the reviews has been combined into one paragraph |
| 02 | 1. All mathematical notations should be consistent. For example, i) Section 3, lines 2 and 3: What is the difference between italic and nonitalic “Y”? ii) Section 3: What is “C Normal”?, iii) All mathematical symbols should be written using equation editor. For example, n=5. Similar comments applied to other sections. | All mathematical notations has been written in italic font uniformly. “C Normal”has been corrected,  All symbols has been written using equation editor. |
| 03 | 1. Section 4, line 3: Spacing between theta_hat and chart. | Space has been given in the the mentioned line. |
| 04 | Incomplete sentences: i) Section 4: “Table 3 and Table 4… [40]”, ii) Section 4: “The results of CUSUM… [31]”, iii) Section 5.1: The ARL1… charts work well,”. Similar comments applied to other sections. | These sentences has been corrected. |
| 05 | 1. Some special names do not need to capitalize it. For example, i) “Average Run Length (ARL)”-> “average run length (ARL)”, ii) “Modified Trimmed Standard Deviation (MTSD)” -> “modified trimmed standard deviation (MTSD)”, iii) “( IQR)” -> non italic “(IQR)” and etc. | The suggestion has been incorporated. |
| 06 | The authors should use a consistent font, italic and nonitalic text. | All abbreviations has been written in italic font. |
| 07 | All abbreviations should be defined for the first time appear in the text. For example, EWMA. | The correction has been incorporated. |
| 08 | 1. Based on the above comments, the authors require to check the manuscript thoroughly | The manuscript has been checked thoroughly for the corrections. |
