# Supplementary material for: A robust cusum control chart for median absolute deviation based on trimming and winsorization
Source: PLoS One. 2024 May 29;19(5):e0297544. doi: 10.1371/journal.pone.0297544 (PMC11135777; doi:10.1371/journal.pone.0297544)
Supplement: Attachment — Submitted filename: fourth Rebuttal letter.docx [file pone.0297544.s009.docx]

**Fourth Rebuttal letter for the Third revision of paper to PLOS ONE Journal**

Dr. Umair Khalil
Academic Editor PLOS ONE & Associate Professor
Department of Statistics,

Abdul Wali Khan University Mardan, Pakistan

September 22, 2023

Dear Dr. Kok Haur Ng
Academic Editor
PLOS ONE

We are thankful to the reviewers for their valuable comments and suggestions on our manuscript title “A Robust Cusum Control Chart for Median Absolute Deviation based on Trimming and Winsorization”. We have revised the manuscript to address their comments.

The point to point response to the comments of reviewer is as under:

| S.No | Comments of the reviewer | Response |
| --- | --- | --- |
| 01 | Section 1: Instead of discussing the reviews one by one in different paragraphs, the authors should combine those similar topics into the same paragraph. | The literature that has been discussed in section 1 has different approaches and different ideas which are included in the references. All those topics which are referred to in section 1 are relevant to the manuscript. The topics which were similar in section 1 are removed. Reference which was on serial Number [11], [20], [21], [23], [24] and [26] in the old manuscript were removed from the revised version. |
| 02 | 1. Incomplete sentences: i) Section 1: “… qualitatively similar The rest…”, ii) Section 3: “…contamination with sample sizes n=5,6 and. For Gamma distribution…”. Similar comments applied to other sections | The mentioned sentences has been corrected in section 1 and section 3. We checked the whole manuscript with software to correct all the incomplete sentences and has corrected the incomplete sentences in the revised version. |
