# Supplementary material for: A robust cusum control chart for median absolute deviation based on trimming and winsorization
Source: PLoS One. 2024 May 29;19(5):e0297544. doi: 10.1371/journal.pone.0297544 (PMC11135777; doi:10.1371/journal.pone.0297544)
Supplement: Attachment — Submitted filename: fifth Rebuttal letter.docx [file pone.0297544.s010.docx]

**Fifth Rebuttal letter for the fifth revision of paper to PLOS ONE Journal**

Dr. Umair Khalil
Academic Editor PLOS ONE & Associate Professor
Department of Statistics,

Abdul Wali Khan University Mardan, Pakistan

December 13, 2023

Dear Dr. Kok Haur Ng
Academic Editor
PLOS ONE

We are thankful to the reviewers for their valuable comments and suggestions on our manuscript title “A Robust Cusum Control Chart for Median Absolute Deviation based on Trimming and Winsorization”. We have revised the manuscript to address their comments.

The point to point response to the comments of reviewer is as under:

| S.No | Comments of the reviewer | Response |
| --- | --- | --- |
| 01 | Section 1: The authors rearrange this section of similar topics into several paragraphs. | This section has been rearranged. |
| 02 | All abbreviations should be defined for the first time appear in the text. For example, CUSUM, SPRT. | All abbreviations are defined now. |
| 03 | All names should use consistent font type. For example, “CUSUM” and “*CUSUM*”. | All abbreviations are now in italic font. |
| 04 | There are some grammatical errors. For example, "Table3 and Table 4 shows" and "From Table 7 and Table 8 results of ARL and SDRL shows". | The grammatical mistakes has been removed. |
| 05 | Some words and symbols are joining together. For example, "According to Tuprah and Ncube [36] whereY_0=0and the…". | It has been corrected |
| 06 | Some sentences are incomplete due to missing ".". s | Missing "." has been added. |
